# Validation of oligoarrays for quantitative exploration of the transcriptome

**DOI:** 10.1186/1471-2164-9-258

**Published:** 2008-05-30

**Authors:** Vigdis Nygaard, Fang Liu, Marit Holden, Winston P Kuo, Jeff Trimarchi, Lucila Ohno-Machado, Connie L Cepko, Arnoldo Frigessi, Ingrid K Glad, Mark A van de Wiel, Eivind Hovig, Heidi Lyng

**Affiliations:** 1 Department of Tumor Biology, Institute for Cancer Research, Norwegian Radium Hospital, Montebello, Oslo, Norway; 2 PubGene AS, Vinderen, Oslo, Norway; 3 Norwegian Computing Center, Oslo, Norway; 4 Department of Developmental Biology, Harvard School of Dental Medicine, Boston, MA, USA; 5 Laboratory for Innovative Translational Technologies, Harvard School of Dental Medicine, Boston, MA, USA; 6 Decision Systems Group, Brigham and Women's Hospital, Boston, MA, USA; 7 Department of Genetics, Harvard Medical School, Boston, MA, USA; 8 Howard Hughes Medical Institute, Harvard Medical School, Boston, MA, USA; 9 Department of Biostatistics, Institute of Basic Medical Sciences, University of Oslo, Norway; 10 Department of Mathematics, University of Oslo, Norway; 11 Department of Mathematics, Vrije Universiteit Amsterdam, The Netherlands; 12 Department of Pathology, VU University Medical Center, Amsterdam, The Netherlands; 13 Department of Medical Informatics, Institute for Cancer Research, Norwegian Radium Hospital, Montebello, Oslo, Norway; 14 Department of Radiation Biology, Institute for Cancer Research, Norwegian Radium Hospital, Montebello, Norway

## Abstract

**Background:**

Oligoarrays have become an accessible technique for exploring the transcriptome, but it is presently unclear how absolute transcript data from this technique compare to the data achieved with tag-based quantitative techniques, such as massively parallel signature sequencing (MPSS) and serial analysis of gene expression (SAGE). By use of the TransCount method we calculated absolute transcript concentrations from spotted oligoarray intensities, enabling direct comparisons with tag counts obtained with MPSS and SAGE. The tag counts were converted to number of transcripts per cell by assuming that the sum of all transcripts in a single cell was 5·10^5^. Our aim was to investigate whether the less resource demanding and more widespread oligoarray technique could provide data that were correlated to and had the same absolute scale as those obtained with MPSS and SAGE.

**Results:**

A number of 1,777 unique transcripts were detected in common for the three technologies and served as the basis for our analyses. The correlations involving the oligoarray data were not weaker than, but, similar to the correlation between the MPSS and SAGE data, both when the entire concentration range was considered and at high concentrations. The data sets were more strongly correlated at high transcript concentrations than at low concentrations. On an absolute scale, the number of transcripts per cell and gene was generally higher based on oligoarrays than on MPSS and SAGE, and ranged from 1.6 to 9,705 for the 1,777 overlapping genes. The MPSS data were on same scale as the SAGE data, ranging from 0.5 to 3,180 (MPSS) and 9 to1,268 (SAGE) transcripts per cell and gene. The sum of all transcripts per cell for these genes was 3.8·10^5 ^(oligoarrays), 1.1·10^5 ^(MPSS) and 7.6·10^4 ^(SAGE), whereas the corresponding sum for all detected transcripts was 1.1·10^6 ^(oligoarrays), 2.8·10^5 ^(MPSS) and 3.8·10^5 ^(SAGE).

**Conclusion:**

The oligoarrays and TransCount provide quantitative transcript concentrations that are correlated to MPSS and SAGE data, but, the absolute scale of the measurements differs across the technologies. The discrepancy questions whether the sum of all transcripts within a single cell might be higher than the number of 5·10^5 ^suggested in the literature and used to convert tag counts to transcripts per cell. If so, this may explain the apparent higher transcript detection efficiency of the oligoarrays, and has to be clarified before absolute transcript concentrations can be interchanged across the technologies. The ability to obtain transcript concentrations from oligoarrays opens up the possibility of efficient generation of universal transcript databases with low resource demands.

## Background

Genomic advances, particularly in sequencing projects, have fueled the progressive development of high throughput technologies for measurement of transcript abundance. The most frequently used techniques are the gene expression microarrays [1], serial analysis of gene expression (SAGE) [2], and massively parallel signature sequencing (MPSS) [3]. There are weaknesses and strengths associated with each of the technologies, and the choice of method depends on the problem to be solved. MPSS and SAGE rely on open-based sampling of transcripts, allowing for the identification of novel transcribed sequences. The complexity of these methods has, however, limited their utility. The less resource demanding and more routinely used microarray platform is a hybridization-based, closed system where the transcript information is restricted to pre-selected probes immobilized on the array [1]. The technologies complement each other and are useful for different purposes, implying that the ability to interchange data across them can be of high value [4]. Hence, large amounts of data that are generated with these techniques and accumulated in publicly available repositories could potentially be merged to create transcript databases of various tissues and used for validation and meta-study purposes. However, before the repositories can be fully utilized in this way, the consistency in the measurements on an absolute scale has to be verified. To our knowledge this has not been done so far, probably due to the lack of a common measurement unit that enables direct comparisons across the technologies.

SAGE and MPSS provide absolute transcript abundance through transcript sampling, sequencing, and identification. They both identify and quantify the transcripts through the generation of short sequence tags (10–22 bp) from the mRNA molecules and present the data as tag counts, facilitating comparisons across these techniques. Tags generated by SAGE are concatemerized and cloned into vectors for conventional dideoxy-sequencing [2]. In MPSS the tags are amplified, loaded onto a microbead library, and immobilized in a flow cell for automated highly-parallel sequencing [3]. Quantification is obtained by counting the frequency of each of the tag sequences in the library, followed by a mapping procedure, which annotates tag to gene. An advantage of MPSS compared to SAGE is the larger library size obtainable when using the same number of sequencing runs [5]. Moreover, the MPSS tags are generally longer, conferring higher specificity with respect to tag annotation.

The microarray technique uses the signal intensity of each array probe as a measure of the mRNA level [1]. There are three major platforms, spotted cDNA arrays, spotted oligoarrays, and *in situ*-synthesized oligoarrays [6]. The data from the spotted arrays are generally presented as the intensity ratio between two samples hybridized together, whereas for *in situ*-synthesized oligoarrays the intensities *per se *have also been used. A fairly good concordance between the data achieved from the different platforms has been demonstrated [7,8]. However, the relative quantification format makes a direct comparison of microarray data with results from other techniques difficult.

Methods to estimate absolute transcript concentrations from microarray data that may be useful for comparisons across technologies have been proposed [9,10]. The TransCount method developed by Frigessi *et al*. [10] is based on Bayesian statistical modeling and utilizes covariates of the microarray experiment to calculate the concentration from the signal intensity of each probe on spotted arrays. The concentration estimate seems to be a more reliable measure of the transcript abundance than the expression ratio usually derived [10]. We have previously applied the method to determine the number of transcripts needed prior to mRNA amplification to obtain reliable expression data from a limited sample quantity [11]. The use of TransCount and the absolute transcript concentrations provides a unique opportunity to explore the consistency in the data achieved with spotted microarrays, MPSS, and SAGE on an absolute scale.

In a recent study we explored the correlations between microarray and MPSS data on a relative scale [4]. The limitations inherent to these technologies were discussed as reasons for the reduced consistency across the technologies compared to within the different hybridization-based techniques. In the present work, we have applied TransCount and estimated transcript concentrations from oligoarray intensities of adult mouse retina, in order to investigate whether the spotted oligoarray technique could provide data that correlated with and had the same scale as those obtained with MPSS and SAGE. We performed a direct comparison between the data sets by converting the transcript concentrations and tag counts to the number of transcripts per cell for each individual gene. Our data suggest that the oligoarrays and TransCount can be used as a substitute to the tag-based techniques for a quantitative exploration of the transcriptome provided that a discrepancy in the absolute scale across the technologies is clarified.

## Results

### Transcript concentrations in adult mouse retina

**Figure 1:**
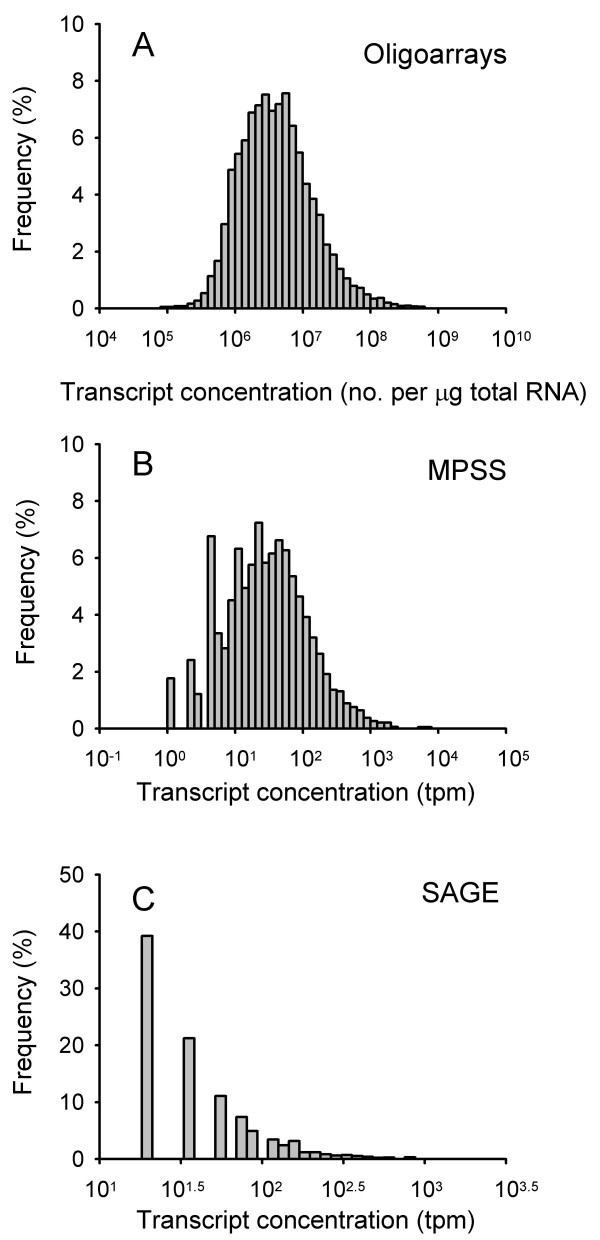
**Frequency distributions of the transcript concentrations measured in adult mouse retina by oligoarrays (A), MPSS (B), and SAGE (C).** The histograms are based on the 9,786 (A), 6,088 (B) and 4,827 (C) unique transcripts detected with each technology.

Oligoarray transcript data were achieved for 14,045 of the 14,076 array probes, showing signal intensities within the whole detection range. The probes were mapped to 9,786 unique UniGene identification numbers (IDs). Transcripts were detected for all UniGene IDs, with concentration ranging over five orders of magnitude (Table 1, Figure 1A). The total transcript concentration for the 9,786 genes was estimated to 1.1·10^11 ^transcripts per μg total RNA, corresponding to 1.1·10^6 ^transcripts per cell and an average value of 112 (range 0.3 – 14,387) transcripts per cell and gene (Table 1).

**Table 1:** Transcript data of adult mouse retina obtained with high throughput technologies

	**Oligoarrays**	**MPSS**	**SAGE**
**All detected genes**			
			
No. of unique transcripts^1^	9,786	6,088	4,827
Total transcript concentration^2^	1.1·10^11 ^(no. per μg total RNA)	5.6·10^5 ^(tpm)	7.6·10^5 ^(tpm)
Average transcript concentration (range)^3,4^	1.1·10^7 ^(3.1·10^4 ^– 1.4·10^9^) (no. per μg total RNA)	92 (1 – 22,008) (tpm)	77 (18 – 6,480) (tpm)
Total no. transcripts per cell^2,3^	1.1·10^6^	2.8·10^5^	3.8·10^5^
Average no. of transcripts per cell and gene (range)^3,4^	112 (0.3 – 14,387)	46 (0.5 – 11,004)	38 (9 – 3,240)

**Overlapping genes**			
			
No. of unique transcripts^1^	1,777	1,777	1,777
Total transcript concentration^2^	3.8·10^10 ^(no. per μg total RNA)	2.2·10^5 ^(tpm)	1.5·10^5 ^(tpm)
Average transcript concentration (range)^3,4^	2.1·10^7 ^(1.6·10^5 ^– 9.7·10^8^) (no. per μg total RNA)	126 (1 – 6,360) (tpm)	86 (18 – 2,536) (tpm)
Total no. transcripts per cell^2,3^	3.8·10^5^	1.1·10^5^	7.6·10^4^
Average no. of transcripts per cell and gene (range)^3,4^	213 (1.6 – 9,705)	63 (0.5 – 3,180)	43 (9 – 1,268)

^1 ^Mapped to UniGene identifiers.

^2 ^Sum for all genes.

^3 ^10 pg total RNA per cell was assumed for oligoarrays, and a total number of 5·10^5 ^transcripts per cell was applied for SAGE and MPSS in the calculation.

^4 ^Average and range for all genes.

MPSS had 6,572 signatures that were reliably mapped to UniGene IDs, out of a total of 34,341 unique tags detected in our library. Among the tags filtered out, 647 were suspected to be in repeated regions, 6,044 had hits on the genomic sequence, but not within transcripts, 5,415 hit the reverse strand, and 14,432 hit the transcripts either without known orientation or without annotated poly(A) tail or polyadenylation signal. There was also a remaining small fraction (4.4%) of signatures that produced no sequence match, most likely attributable to sequencing errors. The 6,572 reliable signatures were mapped to 6,088 unique UniGene IDs with a tag count ranging over four orders of magnitude (Table 1, Figure 1B). The sum of tag counts for all genes was 5.6·10^5 ^tpm, leading to 2.8·10^5 ^transcripts per cell and on average 46 (range 0.5 – 11,004) transcripts per cell and gene (Table 1).

Our SAGE library contained 12,588 unique tags, which were mapped to 4,827 unique UniGene IDs with a tag count ranging over less than three orders of magnitude (Table 1, Figure 1C). A total of 999 of these were UniGene clusters and included more than one tag. The sum of tag counts for 4,827 genes was 7.6·10^5 ^tpm, the number of transcripts per cell was 3.8·10^5^, and the average number of transcripts per cell and gene was 38 (range 9 – 3,240) (Table 1).

### Cross-platform correlations

**Figure 2:**
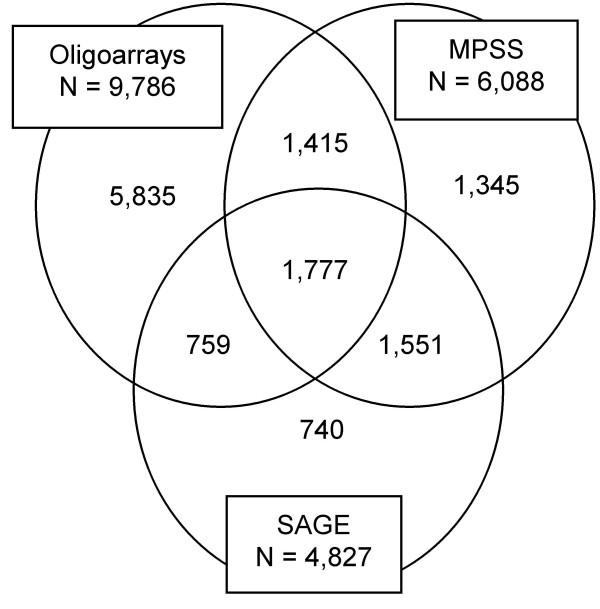
**Venn diagram showing the number and overlap in unique transcripts detected in adult mouse retina by oligoarrays, MPSS, and SAGE.** N is total number of unique transcripts detected with the respective technologies.

**Figure 3:**
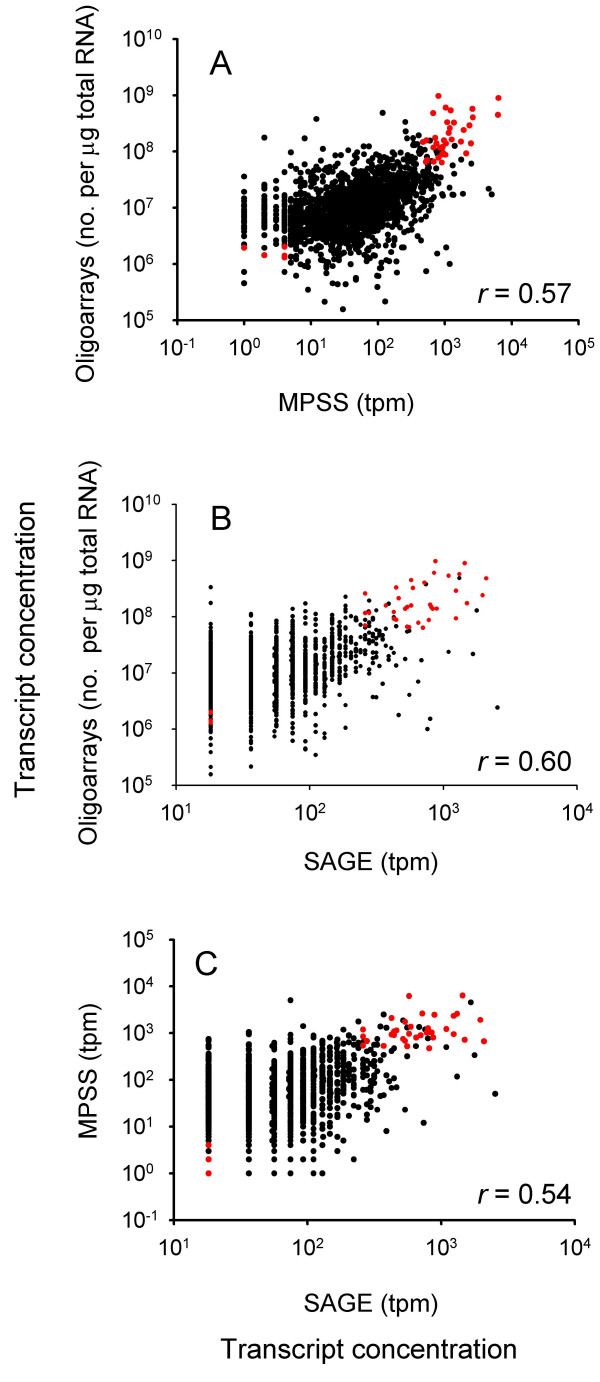
**Comparison of transcript concentrations measured in adult mouse retina with oligoarrays and MPSS (A), oligoarrays and SAGE (B), and MPSS and SAGE (C).** Data for 1,777 overlapping genes are shown on a logarithmic scale. Each dot represents the data of a single gene. The Pearson product moment correlation coefficients, *r*, are indicated (*p *< 0.0001 for all). The corresponding coefficients based on the log transformed data were 0.46 (A), 0.48 (B), and 0.48 (C) (*p *< 0.0001 for all). The 40 transcripts in common for all three technologies among two subsets of 100 genes each, one with the highest and another with the lowest transcript concentrations, are indicated by red dots. The correlation coefficient of the 35 transcripts with the highest concentrations were 0.49, *p *= 0.003 (A), 0.41, *p *= 0.01 (B), 0.22, *p *= 0.2 (C). Analysis of log transformed data showed correlation coefficients of 0.51, *p *= 0.002 (A), 0.45, *p *= 0.007 (B), and 0.30, *p *= 0.08. Number of transcripts per cell for these genes is listed in Table 2.

The three data sets were matched pair-wise according to the UniGene IDs. The number of genes in common for oligoarrays and MPSS was 3,192, while 2,536 and 3,328 genes overlapped between oligoarrays and SAGE, and between MPSS and SAGE, respectively (Figure 2). A subset of 1,777 genes was identified in all three data sets, showing transcript concentrations in the range of 1.6·10^5 ^– 9.7·10^8 ^transcripts per μg total RNA (oligoarrays), 1 – 6,360 tpm (MPSS), and 18 – 2,536 tpm (SAGE) (Table 1, Additional file 1). Comparison of the oligoarray and MPSS data, oligoarray and SAGE data, and MPSS and SAGE data of the 1,777 overlapping genes showed similar relationship with correlation coefficients within the range of 0.54 – 0.60 (Figure 3). The corresponding coefficients based on log transformed data were somewhat lower and ranged from 0.46 – 0.48, showing that the highest transcript concentrations contributed considerably to the correlations of the untransformed data.

**Figure 4:**
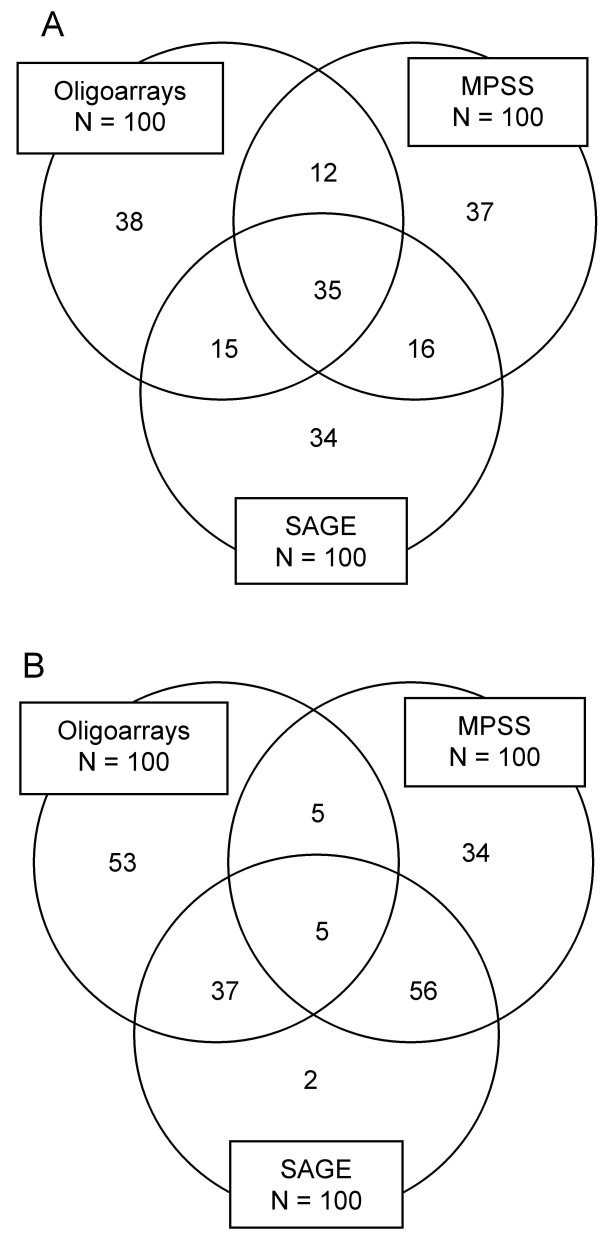
**Venn diagram showing the number and overlap in unique transcripts detected in adult mouse retina by oligoarrays, MPSS, and SAGE.** In (A) the 100 most abundant transcripts for each technology were considered, whereas in (B) the 100 transcripts with the lowest concentration were selected. Number of transcripts per cell for those held in common for all technologies is listed in Table 2.

To further explore the consistency in the data at different transcript concentrations, for each technology we considered two subsets of 100 genes each, one with the highest and another with the lowest concentrations, selected from the data sets of the 1,777 overlapping genes. The expression level of the poorly and highly expressed genes was confirmed by qRT-PCR analysis (Table 2). At the highest transcript concentration, 35 of 100 genes were in common to all technologies, whereas only 5 genes overlapped at the lowest concentration (Figure 4). Similar patterns of intersection were found when more genes were considered (data not shown), showing increased consistency at high concentrations. Although the concentration range of the 35 most abundant transcripts held in common was narrow, their oligoarray data were significantly correlated to the MPSS and SAGE data (Figure 3). The MPSS and SAGE data were not significantly correlated.

**Table 2:** Transcript concentration of selected genes in adult mouse retina^1 ^

**UniGene ID**	**Gene Symbol**	**Gene name**	**Oligoarray**	**MPSS**	**SAGE**	**qRT-PCR**
**Genes with high expression**						
				
Mm.371592	Ubb	Ubiquitin B	9705	401	435	NA
Mm.368524			8916	3180	722	NA
Mm.47709	Pdc	Phosducin	6027	516	425	10.36
Mm.151562	Rbp3	Retinol binding protein 3	5733	1292	657	4.77
Mm.16224	Guca1a	Guanylate cyclase activator 1a (retina)	5366	611	546	0.66
Mm.284811	Unc119	Unc-119 homolog (C.elegans)	4812	335	1046	2.40
Mm.59151	Guca1b	Guanylate cyclase activator 1B	4478	3116	287	1.44
Mm.235863			4046	1312	361	NA
Mm.285993	Calm1	Calmodulin 1	3304	543	222	3.09
Mm.28643	Vamp2	Vesicle-associated membrane protein2	3250	683	296	2.63
Mm.16831	Ckb	Creatine kinase, brain	2896	1164	620	NA
Mm.223674	Syp	Synaptophysin	2592	595	130	0.16
Mm.1372	Pde6b	Diesterase 6B, cGMP, rod reseptor, beta polypeptide	2400	956	982	1.73
Mm.156506	Pcdh21	Protocadherin 21	2137	570	232	1.80
Mm.297482	Tpt1	Tumor protein, translationally controlled 1	1742	359	750	1.44
Mm.3667	Vtn	Vitronectin	1630	634	398	1.74
Mm.352239	Gpsn2	Glycoprotein, synaptic 2	1579	484	287	0.02
Mm.55143	Dkk3	Dickkopf homolog 3 (Xenopus laevis)	1576	266	185	0.10
Mm.726	Bsg	Basigin	1490	870	268	0.87
Mm.683			1472	236	407	NA
Mm.28147	Reep6	Receptor accessory protein 6	1394	1229	445	3.42
Mm.273538		cDNA, clone Y1G0119M19	1389	375	259	NA
Mm.16228	Slc25a4	Solute carrier family 25, member 4	1382	520	416	0.67
Mm.235204	Atp1b2	ATPase, Na+/K+ transporting, beta 2 polypeptide	1210	466	213	1.79
Mm.94160	Bex2	Brain expressed X-linked 2	1171	339	139	0.09
Mm.331	Ubc	Ubiquitin C	1165	423	130	NA
Mm.26633	Plekhb1	Pleckstrin homology domain containing, family B member 1	931	474	620	0.32
Mm.305152	Apoe	Apolipoprotein E	920	1046	213	0.25
Mm.200608	Clu	Clusterin	885	456	222	0.07
Mm.278865	Stxbp1	Syntaxin binding protein 1	873	512	389	0.60
Mm.236513	Pcbp2	Poly(rC) binding protein 2	777	401	325	1.78
Mm.41926			698	271	130	NA
Mm.373613			661	340	268	NA
Mm.379381			650	261	278	NA
Mm.197518	Laptm4b	Lysosomal-associated protein transmembrane 4B	634	451	352	0.30
**Genes with low expression**						
				
Mm.24678	Pard6g	Par-6 partitioning defective 6 homolog gamma (C. elegans)	13	2	9	0.01
Mm.273155	2610110G12Rik	RIKEN cDNA 2610110G12 gene	14	2	9	0.00
Mm.1249	Lamc1	Laminin, gamma 1	14	1	9	NA
Mm.261831			19	1	9	NA
Mm.257952	Tmem159	Transmembrane protein 159	20	2	9	0.01

^1^The 40 transcripts in common for oligoarrays, MPSS and SAGE among two subsets of 100 genes each, one with the highest and another with the lowest transcript concentrations. The values are listed as number of transcripts per cell for oligoarrays, MPSS and SAGE, and as relative to the endogenous control β-actin (*Actb*) for qRT-PCR. NA means not analysed.

### Absolute scale comparisons

Transforming the data to numbers of transcripts per cell and gene allowed us to compare the absolute scale of the measurements for each individual gene across the technologies, applying the three data sets of the 1,777 overlapping genes. The oligoarray values ranged from 1.6 to 9,705 transcripts per cell and gene and were significantly higher than the MPSS and SAGE values (p < 0.001, Friedman test in ANOVA on ranks), which ranged from 0.5 to 3,180 (MPSS) and from 9 to 1,268 (SAGE) transcripts per cell and gene (Table 1, Additional file 1). Hence, *Ubb*, showing the maximum number of transcripts per cell based on oligoarrays (9,705) had only 401 and 435 transcripts per cell based on MPSS and SAGE, respectively (Table 2). More consistent results were, however, achieved for other genes, like *Plekhb1 *(931, 474, 620 transcripts per cell) and *Laptm4b *(634, 451, 352 transcripts per cell) (Table 2). The average number of transcripts per cell was 213 (oligoarrays), 63 (MPSS), and 43 (SAGE).

The absolute transcript concentrations also enabled us to compare the detection efficiency at different transcript concentrations among the technologies. For the 35 most abundant transcripts held in common, a total number of 89891, 25687, and 13655 transcripts per cell were detected with the oligoarray, MPSS, and SAGE technique, respectively (Table 2). Assuming that all these were true transcripts, MPSS detected 29% and SAGE 15% of those detected with oligoarrays. For the 5 overlapping genes at low transcript concentration, MPSS detected 8 (10%) and SAGE 45 (56%) of the totally 80 transcripts detected with oligoarrays (Table 2). The median detection efficiency based on all overlapping genes was 21% (MPSS) and 23% (SAGE), as compared to the oligoarray data. The oligoarrays therefore seemed to be more sensitive in detecting known transcripts.

## Discussion

The use of TransCount to retrieve absolute units from oligoarray data in our study enabled a quantitative comparison of transcript concentrations across MPSS, SAGE, and spotted oligoarrays. Although several studies have compared the performance of tag-based and hybridization-based gene expression platforms [4,12-19], our focus on a common measurement unit has to our knowledge not received detailed attention so far. Previous comparisons involving microarrays have utilized the signal intensities [4,12-19]. The intensities of *in situ*-synthesized oligoarrays may possibly reflect the transcript concentration reasonably well, but intensities are not suitable when spotted arrays are used and not directly comparable across experiments and platforms. By our approach, the numbers of transcripts per cell were calculated genome-wide for all three technologies. These values could be compared directly across the technologies, and a thorough validation of oligoarrays for quantitative exploration of the transcriptome could be performed.

Our study did not allow for a general evaluation of the transcriptome coverage of each platform, since differences in the sampling depth among the technologies would bias the outcome. Given our sampling depths of 1.6 million and 55,000, which are commonly used in MPSS and SAGE experiments respectively, only about 60% (MPSS) and 10% (SAGE) of the transcripts with 1–5 copies per cell are expected to be detected [20]. Hence, to identify 90% of the expressed genes, sequencing of about three million tags is probably required [20], leading to a significant increase in the costs of these experiments. In contrast, transcriptome coverage in the oligoarray data was more explicitly defined and easier to be ensured by performing four replicate experiments. Rapid advances in the development of next generation sequencing technologies may eventually fill this gap by allowing for significant improvements in the sampling depth at dramatically reduced cost and time. On the other hand, the aim of this study was to validate the quantitative potential of the oligoarrays. We therefore focused primarily on the subset of genes detected by all three technologies, through a stringent mapping of MPSS and SAGE tags to known genes, with a further limit to those also present in the oligoarray design. This explains why the number of unique transcripts detected was lower for the tag-based techniques than for the oligoarrays. Although the increase in sampling depth of tag-based technologies may considerably facilitate better transcriptome coverage, it is not a crucial concern in our study.

The oligoarray estimates showed a stronger relationship to the MPSS and SAGE data than that previously reported for spotted oligoarrays [13], possibly because we used absolute transcript concentrations and not intensities in the analysis. Hence, we have previously shown that the absolute transcript concentrations derived from TransCount is more strongly correlated to qRT-PCR data than are the relative values achieved from traditional microarray analysis, suggesting that they are more reliable measures of the transcript abundance [10]. Otherwise, our results, including the particularly poor correlation at low concentrations, were in agreement with earlier reports [12-19]. A correlation coefficient of about 0.50–0.60 therefore probably reflects the overall consistency across the technologies when genes at all expression levels are included. The correlations involving the oligoarray data were not weaker than, but, similar to the correlation between the MPSS and SAGE data, both when the entire concentration range was considered and at high concentrations. Inherent technological differences in the detection and quantification processes between the techniques probably caused some inconsistency between the data sets. Disadvantages related to the respective technologies, such as cross-hybridization, sampling variances, and tag annotation ambiguities may have contributed [18,19,21]. The discrepancy was therefore probably caused by erroneous measurements in all data sets.

The correlations involving the SAGE data may have been influenced by the use of another RNA pool in these experiments than in the MPSS and oligoarray experiments. Mouse retina generally shows low variability in gene expression, minimizing possible confounding effects caused by differences in the RNA pools. Hence, in a recent study we showed that data variation introduced by biological replicates of the mouse retina is small compared to the variation caused by using different technologies [4]. Moreover, the correlation to oligoarray data was somewhat stronger for the SAGE than the MPSS data. The use of another RNA pool for the SAGE experiments had therefore probably minor influence on our results.

The number of transcripts per cell was considerably higher based on oligoarrays and TransCount than based on MPSS and SAGE, both at high and low concentrations. The difference in the absolute scale of the measurements depends on the values used for the sum of all transcripts and the total RNA content per cell in the MPSS/SAGE and oligoarray calculations, respectively. Our oligoarray results suggest that the maximum number of transcripts may exceed one million, which is more than two-fold higher than the 5·10^5 ^reported in a study from 1976 [22] that was used in our MPSS and SAGE estimations. Adjusting this number to 1.5·10^6 ^transcript per cell would have led to MPSS and SAGE values more comparable to the absolute oligoarray data. Consequently, the calculated transcript detection efficiency will also be more similar for the three technologies. Hence, the transcript detection efficiency seemed to be considerable higher for the oligoarrays when the value of 5·10^5 ^transcript per cell was used in the MPSS and SAGE estimations. More recent studies exploring the number of transcripts in cells have not been performed, except for a microarray study where spike-in controls were used to define a standard curve, which related signal intensity to the absolute transcript numbers [9]. The transcript values were two- to three-fold lower than ours, but the apparent discrepancy was solely due to the use of a highly conservative value of 2–3 pg total RNA per cell in their calculations. Our estimate of 10 pg per cell is within the range of previously reported data [23,24], and probably closer to the true value. The findings reported in the other microarray study [9] are therefore in agreement with our results. Although the error range of the oligoarray estimates is relatively large [10] and the data may be somewhat overestimated, due to possible unspecific binding to array probes [25] and experimental uncertainty in the scaling of the absolute values [10], these findings strongly suggest that the value of 5·10^5 ^should be re-examined and probably elevated. MPSS and SAGE would then be found to have a weaker coverage than previously anticipated.

The increased estimate for the sum of all transcripts per cell based on oligoarrays was also reflected in a higher number of transcripts per cell and gene, as compared to the MPSS and SAGE results. A number of about 10,000 transcripts was estimated for several genes, and numbers above 5,000 were found for 15 genes, when considering all the detected transcripts. In contrast, only three genes had a transcript number above 5,000 by MPSS, whereas by SAGE the highest number was 3,240. More than 10,000 transcripts per cell have been reported for individual genes and gene groups in several studies on mouse tissues [20,26-28], consistent with our oligoarray data. Moreover, TransCount estimations for cervical cancers based on cDNA microarrays led to values in agreement with the present oligoarray results [10]. These observations further question the validity of the total transcript number of 5·10^5 ^per cell that was used in the MPSS and SAGE calculations. In that respect, a recent SAGE study showed that using a total number of 1·10^6 ^transcripts per cell to convert the tag counts led to absolute transcript numbers consistent with the published values mentioned above [20], supporting our findings.

A thorough evaluation of genes expressed in adult mouse retina and their putative function have been presented in previous studies based on the SAGE data [29,30]. Here, we focused on 40 genes with particularly high or low expression regardless of technology, suggesting that these are truly up- or downregulated compared to the average expression level. The most abundant transcripts are known to be involved in visual perception (*Pdc*, *Rbp3*, *Guca1a*, *Unc119*, *Guca1b*, *Pde6b*) or play another role in retinal function (*Calm1*, *Syp*) [30]. Moreover, high expression of *Bsg*, *Plekhb1*, *Reep6*, and *Stxbp1 *has been reported in the retina, photoreceptors, and/or eye [31-33]. Our findings are therefore consistent with previous reports and point to more genes that may be explored to increase our understanding of retinal function, like *Ubb *and *Vamp2*. The data also support the hypothesis that the most abundant transcripts are tissue specific and involved in specialized functions, whereas the larger number of less abundant transcripts may be involved in housekeeping activities and shared between tissues, as suggested from studies on the mouse liver [22].

## Conclusion

The transcript concentrations estimated from spotted oligoarrays by use of TransCount are correlated to those obtained with MPSS and SAGE. Oligoarrays and TransCount may therefore play a role in an efficiently building of transcript repositories at low costs and labor demands. Such quantitative data may also enable insight into new aspects of the transcriptome and a better understanding of gene networks [34]. Clarification of the discrepancy in the absolute scale of the measurements would imply that data may be interchanged across hybridization- and tag-based technologies.

## Methods

### Tissue sample

Total RNA from B6 adult mouse retina was used throughout the study. The oligoarray and MPSS experiments were based on the same RNA pool, whereas a different pool was used in the SAGE experiments [29]. Details of the sample collection and RNA extraction can be found in Kuo *et al*. [7]. Quality assessment was performed on a Bioanalyzer (Agilent Technologies, Inc., Santa Clara, CA) to ensure that high quality RNA was used.

### Microarray experiments

Spotted mouse 70-mer oligoarrays produced at the microarray facility at the Norwegian University of Science and Technology were used. The arrays contained 32,448 spots with 14,076 unique probes printed from an oligonucleotide set originating from the Operon mouse oligo collection v3.0 (Operon Biotechnologies, Inc, Huntsville, AL). Control probes from the Spot Report Alien Oligo Array Validation System (Stratagene, La Jolla, CA) were printed 48 times each across the array. The control spots were used by TransCount to find the absolute scale of the transcript concentrations [10]. A self-self hybridization design with 4 array replicates was used.

Cy3 and Cy5 labeled cDNA was synthesized from 13.5 – 15 μg total RNA, as described previously [10]. The quality of the labeled cDNA was assessed from the ratio of absorbance at 260 nm and 280 nm, as measured by use of a NanoDrop spectrophotometer (NanoDrop Technologies, Wilmington, DE). The quality was found to be satisfactory. To target the control probes, 9 control mRNA spikes from the Spot Report system were added to the reaction mixtures at well-defined concentrations, ranging from 3.3 × 10^7 ^to 2.7 × 10^9 ^mRNA molecules. A 25% formamide-based hybridization buffer was added to the labeled target mixture, and the mixture was applied to the array for overnight hybridization at 42°C in a water bath. The slides were scanned by an Agilent G2566AA scanner (Agilent Technologies, Inc., Santa Clara, CA) at two PMT settings of 100 and 50, enabling correction of saturated spot intensities [35] and estimation of the scanner amplification factors needed in TransCount to calculate the transcript concentrations [10].

The TransCount method, originally developed for cDNA microarrays [10], was applied with small modifications for oligoarrays. The sequence length of all probes was 70 bp, and the intensities from a slide stained with SYTO nucleic acid staining dye (Molecular Probes, Inc, Eugene, OR) were used as probe quantities. Since oligoarrays are affected by less experimental variation than cDNA microarrays, TransCount could be directly applied with these modifications [10]. The transcript concentrations were estimated from the saturation and background corrected intensities of each probe and oligoarray. The intensities of the control spots covered the whole detection range from near background values to saturation. The estimated concentrations of these spots showed a highly linear relationship to the true concentrations, suggesting reliable scaling of the concentrations of the other spots. The mean concentrations of the four data sets were used in the further analyses. The average number of transcripts per cell was calculated by assuming a total RNA content of 10 pg per cell [23]. Each probe was assigned a UniGene ID by searching the best sequence match in the mouse UniGene build 151 to the probe sequence. If more than one probe was mapped to the same UniGene ID, their transcript estimates were averaged.

### MPSS

Total RNA samples were sent to Lynx Therapeutics (now Illumina, Hayward, CA) for processing. The MPSS library was generated according to the Megaclone protocol [3]. Signatures adjacent to poly(A) proximal *DpnII *restriction sites, comprising of 20 nucleotides each, including the *DpnII *recognition sequence "GATC", were cloned into a Megaclone vector. The resulting library was amplified and loaded onto microbeads. About 1.6 million microbeads were loaded into a flow cell, and the signature sequences of 17 bases were read out by a series of enzymatic reactions. The abundance of each signature was converted to transcripts per million (tpm).

The mapping of signatures to genes was based on the mouse genome sequence (UCSC GoldenPath genome database, Release 3, Feb 2003) and the UniGene build 151, using the Automatic Correspondence of Tags and Genes (ACTG) tool [36]. A complete set of possible "virtual signatures" was extracted from the sequence database to generate a comprehensive mouse signature collection, and all signatures were ranked and classified according to the likelihood of being a true and detectable signature. If a signature had been located close to a polyadenylation signal or a poly(A) tail on mRNA sequences with known orientation, the credibility of the tag-to-gene assignment was the highest, and the signature was included. In contrast, if a signature was extracted from mRNA sequences whose transcriptional orientation, polyadenylation features, or position information was unknown, or had been found only in non-coding regions or repeated structures, it was filtered out. Such hits may have been generated due to the currently incomplete annotation of the murine genome, or due to sequencing errors in the MPSS experiments. For genes with more than one representative tag sequence, all the corresponding tag counts were summed up. To calculate the average number of transcripts per cell the number of tags per million was divided by two, assuming that the total number of transcripts per cell was 5·10^5 ^[22].

### SAGE

The SAGE data from a previously published study were used [29]. Generation of the SAGE transcript library and the data processing to extract tags and eliminate duplicates have been described earlier [29]. The total number of sequenced tags in the library was about 55,000. The tag counts, originally normalized to 55,000, were converted to tags per million (tpm). The tag-to-gene mapping was performed using the ACTG tool based on a SAGEmap data release with UniGene build 151 [37]. Only the tags that had a reliable tag-to-UniGene match in SAGEmap were included for further analyses. For cases where more than one tag was mapped to one gene, the tag counts were pooled in the same manner as for the MPSS data. The average number of transcripts per cell was calculated as for the MPSS data.

### Quantitative real-time PCR

We used qRT-PCR to confirm the mRNA levels of 28 genes listed in Table 2. Our criteria for designing the primers included that they were intron-spanning. This was not the case for 7 of the 35 genes in Table 2, and these were therefore not analysed. QRT-PCR was applied, using Roche 480 LightCycler. Mouse Universal ProbeLibrary probes and target-specific PCR primers (Additional file 2) were selected using the ProbeFinder assay design software [38]. All assays were prepared using standard conditions in a master mix solution (Roche Applied Sciences). cDNA was synthesized from 10 μg of total RNA for each sample using Roche reverse-transcriptase. The reactions were run in triplicate for each gene, using 20 μl reaction volumes and the following conditions: 95°C for 5 minutes, 45 cycles for 95°C for 10 seconds, 60°C for 15 seconds, and 72°C for one second. Dilution curves were made to ensure appreciable amplification efficiency (Additional file 3). The transcript concentrations were calculated relative to the endogenous control β-actin (*Actb*) as $2^{-(Ct_{Gene}-Ct_{Actb})}$, where *Ct*_*Gene *_and *Ct*_*Actb *_correspond to the mean cycle thresholds for the test gene and β-actin, respectively [39].

#### Array express accession

The raw data from the oligoarray platform have been deposited to the Array Express repository (E-TABM-422).

## Authors' contributions

VN, FL, MH, AF, IKG, EH, and HL conceived and designed the study. VN and FL performed the microarray experiments and matched the data from the different techniques. VN, FL and HL wrote the article. MH, AF, IKG, MAvdW, and HL contributed to the Transcount analysis. WPK, JT, LO-M, and CLC provided the SAGE, MPSS, and qRT-PCR data. All authors helped to draft the manuscript and read and approved the final version.

## Supplementary Material

Additional file 1 Transcript data of genes in common for oligoarrays, MPSS, and SAGE. The file lists transcript data obtained with oligoarrays (number of transcripts per μg total RNA and number of transcripts per cell), MPSS (tags per million and tags per cell) and SAGE (tags per million and tags per cell) for the overlapping genes. Click here for file

Additional file 2 QRT-PCR primer sequences, The file lists the forward and reverse primer sequences and amplicon sequences for the qRT-PCR analyses. Click here for file

Additional file 3 PCR amplification efficiency of selected genes. The file lists the slope and standard deviation of linear curves fitted to plots of cycle threshold (Ct) *versus *primer dilution for the genes subjected to qRT-PCR analysis. Click here for file
